# Variations in the Consumption of Antimicrobial Medicines in the European Region, 2014–2018: Findings and Implications from ESAC-Net and WHO Europe

**DOI:** 10.3389/fphar.2021.639207

**Published:** 2021-06-17

**Authors:** Jane Robertson, Vera Vlahović-Palčevski, Kotoji Iwamoto, Liselotte Diaz Högberg, Brian Godman, Dominique L. Monnet, Sarah Garner, Klaus Weist, ESAC-Net Study Group, Reinhild Strauss, WHO Europe AMC Network Study Group, Narvina Sinani

**Affiliations:** ^1^ World Health Organization Regional Office for Europe, Copenhagen, Denmark; ^2^ Department of Clinical Pharmacology, The University of Newcastle, Callaghan, NSW, Australia; ^3^ Department of Clinical Pharmacology, University Hospital Rijeka/Medical Faculty and Faculty of Health Studies, University of Rijeka, Rijeka, Croatia; ^4^ European Centre for Disease Prevention and Control, Solna, Sweden; ^5^ Strathclyde Institute of Pharmacy and Biomedical Sciences, University of Strathclyde, Glasgow, United Kingdom; ^6^ Department of Public Health Pharmacy and Management, School of Pharmacy, Sefako Makgatho Health Sciences University, Pretoria, South Africa

**Keywords:** antibiotic utilization, antimicrobial medicines consumption, AWaRe classification, cross-national comparative study, drug utilization 75%, European Surveillance of Antibiotic Consumption Network, Eastern Europe, Central Asia

## Abstract

**Background:** Surveillance of antimicrobial consumption (AMC) is important to address inappropriate use. AMC data for countries in the European Union (EU) and European Economic Area (EEA) and Eastern European and Central Asian countries were compared to provide future guidance.

**Methods:** Analyses of 2014–2018 data from 30 EU/EEA countries of the European Surveillance of Antibiotic Consumption network (ESAC-Net) and 15 countries of the WHO Regional Office for Europe (WHO Europe) AMC Network were conducted using the Anatomical Therapeutic Chemical (ATC) classification and Defined Daily Dose (DDD) methodology. Total consumption (DDD per 1000 inhabitants per day) of antibacterials for systemic use (ATC group J01), relative use (percentages), trends over time, alignment with the WHO Access, Watch, Reserve (AWaRe) classification, concordance with the WHO global indicator (60% of total consumption should be Access agents), and composition of the drug utilization 75% (DU75%) were calculated.

**Findings:** In 2018, total consumption of antibacterials for systemic use (ATC J01) ranged from 8.9 to 34.1 DDD per 1000 inhabitants per day (population-weighted mean for ESAC-Net 20.0, WHO Europe AMC Network 19.6, ESAC-Net Study Group, and WHO Europe AMC Network Study Group). ESAC-Net countries consumed more penicillins (J01C; 8.7 versus 6.3 DDD per 1000 inhabitants per day), more tetracyclines (J01A; 2.2 versus 1.2), less cephalosporins (J01D; 2.3 versus 3.8) and less quinolones (J01M; 1.7 versus 3.4) than WHO Europe AMC Network countries. Between 2014 and 2018, there were statistically significant reductions in total consumption in eight ESAC-Net countries. In 2018, the relative population-weighted mean consumption of Access agents was 57.9% for ESAC-Net and 47.4% for the WHO Europe AMC Network. For each year during 2014–2018, 14 ESAC-Net and one WHO Europe AMC Network countries met the WHO global monitoring target of 60% of total consumption being Access agents. DU75% analyses showed differences in the choices of agents in the two networks.

**Interpretation:** Although total consumption of antibacterials for systemic use was similar in the two networks, the composition of agents varied substantially. The greater consumption of Watch group agents in WHO Europe AMC Network countries suggests opportunities for improved prescribing. Significant decreases in consumption in several ESAC-Net countries illustrate the value of sustained actions to address antimicrobial resistance.

## Introduction

Antimicrobial resistance (AMR) is one of the main public health threats worldwide. It is estimated that each year about 33,000 deaths are attributable to infections with antibiotic resistant bacteria in the European Union (EU)/European Economic Area (EEA) (Cassini et al., 2019) and 700,000 globally (O'Neill, 2016). The Organization for Economic Co-operation and Development (OECD) predicts that the costs associated with AMR could increase to US$ 3.5 billion per year if not addressed (Hofer, 2019).

However, the problem of AMR calls for concerted efforts at the local, regional and national level as well as close international cooperation which is not always forthcoming (Laxminarayan et al., 2020). Ensuring prudent antimicrobial use is a key priority in an effective response to AMR, as antimicrobial use exerts ecological pressure on bacteria and contributes to the emergence and selection of resistant bacteria. The importance of surveillance of antibiotic consumption to identify potential overuse, underuse and inappropriate use is highlighted in the World Health Organization (WHO) Global Action Plan on AMR (World Health Organization, 2015), as well as the WHO European Strategic Action Plan on Antibiotic Resistance (World Health Organization Regional Office for Europe, 2011) and the European One Health Action Plan against AMR (European Commission, 2017).

There are two main networks in the WHO European Region that collate data on consumption of antimicrobial medicines. The European Surveillance of Antimicrobial Consumption Network (ESAC-Net) coordinated by the European Centre for Disease Prevention and Control (ECDC) collates and analyses data on antibiotic consumption from 30 countries in the EU/EEA (European Centre for Disease Prevention and Control, 2020). Data are available since 1997 and are published on-line and in annual reports. The WHO Regional Office for Europe (WHO Europe) Antimicrobial Medicines Consumption (AMC) Network has been undertaking systematic surveillance of antimicrobial medicines consumption since 2011 (World Health Organization Regional Office for Europe, 2020a). AMC data for 2011–2017 for 17 network members have been published (World Health Organization Regional Office for Europe, 2020b).

Both networks use standardized methods to collect and analyze data for total AMC, and where possible, disaggregated to community and hospital AMC. Data collection is based on the WHO Anatomical Therapeutic Chemical (ATC) classification system and Defined Daily Dose (DDD) methodology (WHO ATC/DDD Index, 2018, 2020). Few data have been published comparing AMC data from the two networks. Data on total antibiotic consumption in 2011 and 2015 have been published (Versporten et al., 2014; Robertson et al., 2019). The WHO global report on antimicrobial consumption presented combined European data for 2015 (World Health Organization, 2018).

This study extends these comparisons, comparing data from 30 EU/EEA countries participating in ESAC-Net with data from 15 countries of the WHO Europe AMC Network where Ministries of Health have agreed on data sharing and publication. The aim of this study was to examine total consumption and patterns of consumption of antibiotics, i.e. antibacterials for systemic use (ATC group J01), in DDD per 1000 inhabitants per day. This is the primary indicator of antibiotic consumption in countries as defined by the European Commission and WHO (European Centre for Disease Prevention and Control, European Food Safety Authority and European Medicines Agency (ECDC/EFSA/EMA), 2017a). The relative consumption of oral and parenteral formulations, the comparative use of WHO Access, Watch and Reserve (AWaRe) antibiotics, and concordance with the monitoring indicator that 60% of total consumption should be Access agents (Sharland et al., 2019; Klein et al., 2020; World Health Organization, 2020) were assessed and trends over time examined. In the absence of indication-linked information on antibiotic use, the WHO AWaRe classification allows a more detailed analysis of aggregated data and opportunities for stewardship activities (Sharland et al., 2019). The AWaRe classification has been applied to patterns of total antibiotic consumption globally in 2015 (World Health Organization, 2018), changes over time between 2000 and 2015 (Klein et al., 2020), analyses of pediatric antibiotic sales data (Hsia et al., 2019a), published studies of antibiotic use in low- and middle-income countries (Sulis et al., 2020), analyses of national consumption estimates (McGettigan et al., 2017; Malik and Figueras, 2019; Public Health Agency of Canada, 2020) and patterns of consumption in hospitals (Schweickert et al., 2018; Hsia et al., 2019b).

The combined data presented here provide a pan-European perspective on rates and patterns of AMC between 2014 and 2018.

## Materials and Methods

### Data Collection

**FIGURE 1:**
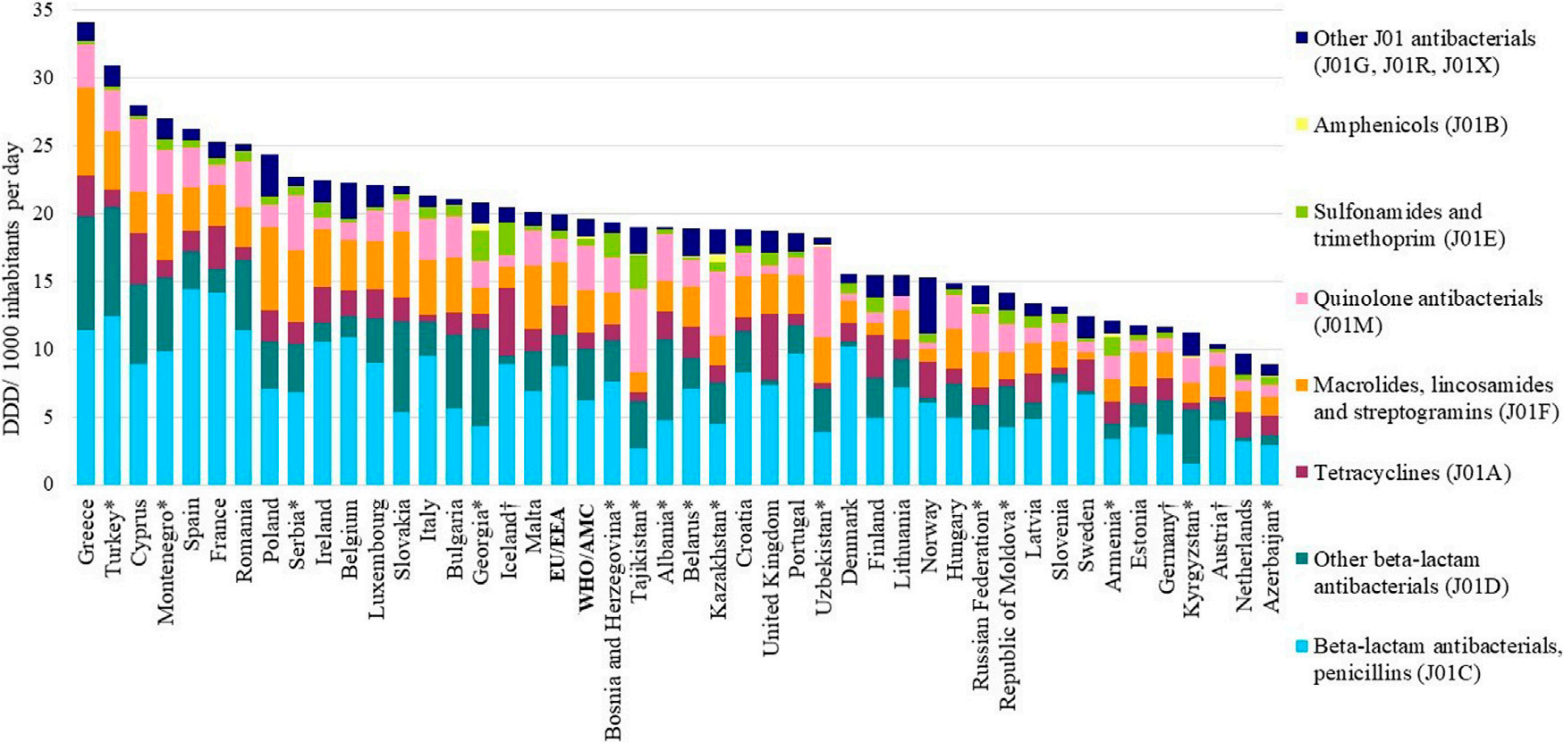
Total consumption of antibacterials for systemic use (ATC J01) expressed in DDD per 1000 inhabitants per day, by pharmacological subgroup, 2018. *Member of the WHO Europe AMC Network. EU/EEA: population-weighted mean for countries of ESAC‐Net. WHO/AMC: population-weighted mean for countries of the WHO Europe AMC Network. ^†^Countries reported only community data.

The data for this study were retrieved from two surveillance networks in Europe – 30 countries of the ESAC-Net and 15 countries of the WHO Europe AMC Network – for the period 2014 to 2018 (data last reviewed on October 1, 2020; Figure 1). Details on the methods of data collection and analysis for both networks have previously been published (European Centre for Disease Prevention and Control, 2020; World Health Organization Regional Office for Europe, 2020b). Briefly, data collection follows standardized protocols and only includes medicines with an assigned ATC code, facilitating analyses from the main classes (ATC level 1) to individual substances (ATC level 5). The main analyses presented here are for the antibacterials for systemic use (ATC group J01). Data on additional antimicrobials are included in the calculation of the WHO Access, Watch and Reserve (AWaRe) classification (World Health Organization, 2019), namely neomycin (A07AA01), streptomycin (A07AA04), polymyxin B (A07AA05), kanamycin (A07AA08), vancomycin (A07AA09), colistin (A07AA10), rifamixin (A07AA11), rifampicin (J04AB02), rifamycin (J04AB03), rifabutin (J04AB04), metronidazole (P01AB01).

### Data Sources

ESAC-Net data are predominantly derived from sales data, reimbursement data or a combination of these sources (Supplementary Table S1A). Reimbursement data do not include consumption of antimicrobials obtained without prescription and other non-reimbursed courses of therapy compared to sales data. The extent of consumption not included when using only reimbursement data may vary by country (Safrany and Monnet, 2012). ESAC-Net data represent total coverage of consumption in the countries except for Germany (85% coverage), the Netherlands (92%) and Luxembourg (91%).

Countries that participate in the WHO Europe AMC Network mostly rely on import data using customs records and declaration forms supplemented with sales records from market authorization holders, local manufacturing estimates, wholesaler records, and in some cases, commercial data sources (Supplementary Table S1B). Data represent total consumption estimates except for Kazakhstan where a commercial data source provides coverage of 80% of hospital and community sales.

Across both networks, there are countries that have changed data sources during the study period and/or where data are not available for all years examined.

### Data Analysis and Metrics Reported

Data disaggregated to community and hospital sector consumption were not available for all studied countries. Consequently, the main analyses presented here are for total consumption across both sectors combined. Total numbers of DDDs for each product were aggregated to give the total number of DDDs at the desired ATC code level and adjusted for the population size to calculate DDDs per 1000 inhabitants per day. For countries where there was incomplete coverage, data were extrapolated to estimate 100% consumption to facilitate comparisons across countries and networks. The ATC/DDD Index 2019 was applied to all years of data to facilitate analysis of trends over time (WHO Collaborating Centre for Drug Statistics Methodology, 2020). Eurostat (European Commission 2020a) or national population estimates were used for EU/EEA countries and World Bank population estimates (The World Bank, 2020) were applied to WHO Europe AMC Network countries apart from Turkey, where estimates were adjusted to take account of the large refugee population. Patterns of consumption of antibacterials for systemic use (ATC group J01) by ATC level 3 sub-groups and by route of administration (oral and parenteral) in 2018 were assessed.

Measures of relative consumption, expressed as a percentage of total consumption of groups of antimicrobials, were derived for 3rd ATC level group of antibacterials for systemic use, and for the WHO AWaRe groups of antibiotics (World Health Organization, 2019). The proportion of total consumption that comprised Access agents was calculated and trends 2014–2018 assessed.

The most frequently used antibiotic substances comprising 75% of the total consumption (the Drug Utilization 75%, DU75%) were derived and stratified by route of administration (Zarb et al., 2011).

Summary data were considered for the networks separately using arithmetic and population-weighted mean estimates. For each network, a population-weighted estimate of total consumption was calculated by multiplying DDD per 1000 inhabitants per day for each country with the corresponding population, then summing the country estimates and finally dividing the resulting total number of DDDs by the total population of all participating countries (European Centre for Disease Prevention and Control, 2019a). Using similar methods, population-weighted estimates were calculated for the relative consumption of AWaRe groups of antibiotics and for components of the DU75%.

Simple linear regression was used to assess trends in antibiotic consumption for each participating country. The output of the software used (Excel data analysis module) includes the regression statistics of a linear regression analysis and the corresponding analysis of variance (ANOVA) test statistics. To illustrate changes in antimicrobial consumption rates over time, we calculated the compound annual growth rate (CAGR) of total antibiotic consumption for each country, which reflects the average annual change as a proportion (%) of the consumption in the starting year. CAGR were estimated for countries that had five years of available data. P values ≤0.05 were considered statistically significant.

### Role of the Funding Source

For ESAC-Net, no specific funding was allocated for this study. Netherlands Ministry of Health, Welfare, and Sport, and the German Collaboration Program provide funding for the WHO Europe AMC Network, but had no role in the study design, data collection, data analysis, data interpretation, or writing of the manuscript.

## Results

**FIGURE 2:**
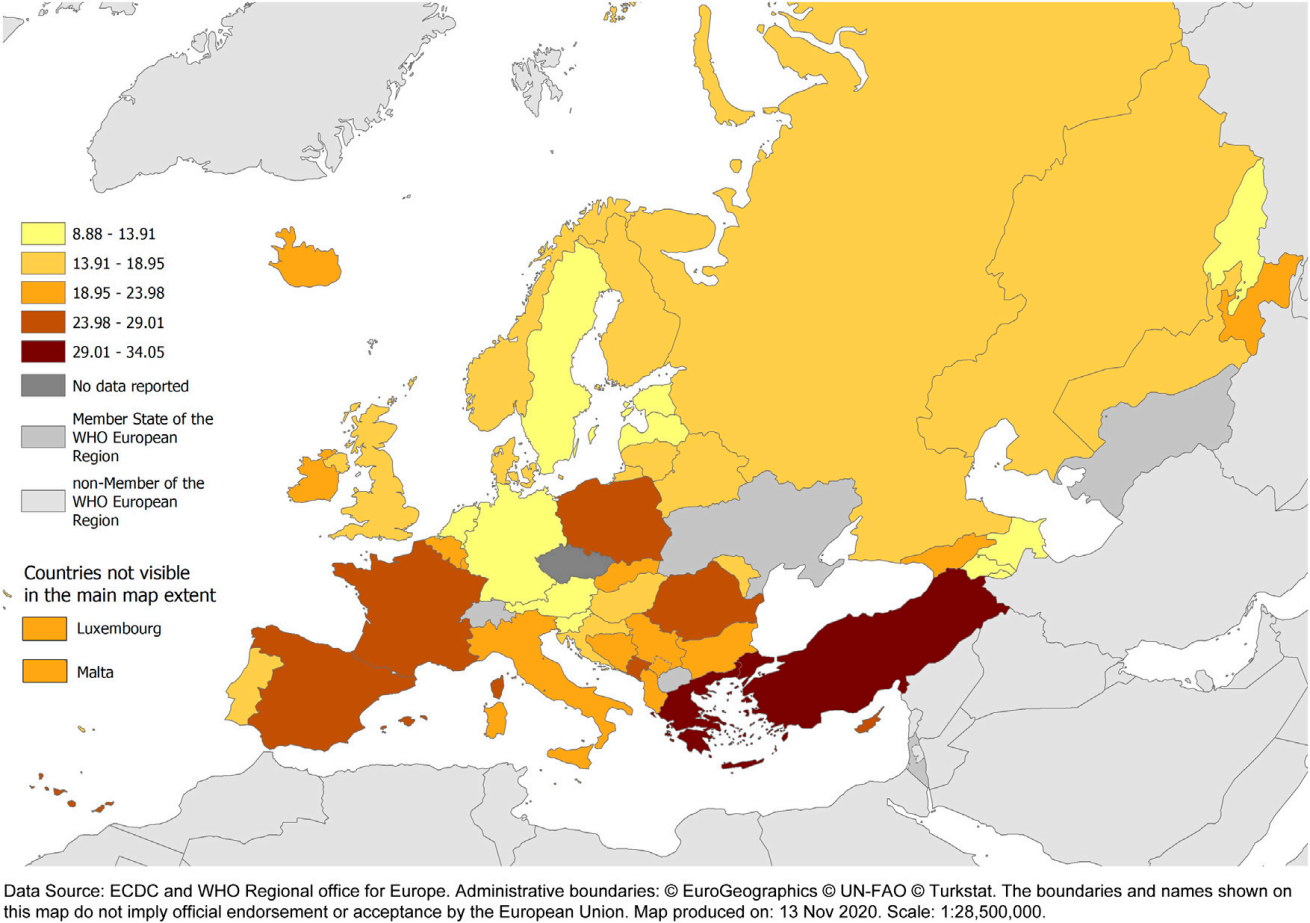
Total consumption of antibacterials for systemic use (ATC J01) (expressed as DDD per 1 000 inhabitants per day) for countries of ESAC‐Net and the WHO/Euro AMC Network, 2018. Austria, Iceland and Germany reported only community data.

The arithmetic mean and the population-weighted mean total consumption of antibacterials for systemic use (ATC group J01) in 2018 were similar in the two networks–19.0 and 20.0 DDD per 1000 inhabitants per day, respectively, for ESAC-Net compared to 18.4 and 19.6 DDD per 1000 inhabitants per day, respectively, for the WHO Europe AMC Network. However, large inter-country variations were seen among both the 29 ESAC-Net countries (9.7–34.1 DDD per 1000 inhabitants per day) and the 15 WHO Europe AMC Network countries (8.9–30.9 DDD per 1000 inhabitants per day) (Figures 1, 2, Supplementary Table S2).

In 2018, the relative consumption of parenteral formulations varied within and between networks. For the 26 ESAC-Net countries for which hospital sector data were included in total consumption of antibacterials for systemic use, parenteral formulations represented from 3.6 to 23.9% of total consumption (median 5.9%). For the 15 WHO Europe AMC Network countries, parenteral formulations comprised between 2.8 and 40.1% (median 13.1%) of total consumption (Supplementary Table S3).

The population-weighted consumption of penicillins (ATC J01C) differed in the two networks – 8.7 versus 6.3 DDD per 1000 inhabitants per day and representing 43.8 and 31.9% of total consumption of antibacterials for systemic use for ESAC-Net and WHO Europe AMC Network countries, respectively (Supplementary Table S2). Penicillins were the most consumed subgroup of antibiotics in 28 (97%) of 29 ESAC-Net countries (range 24.3–65.9% of total consumption) and 9 (67%) of 15 WHO Europe AMC Network countries (range 14.2–40.2%).

In ESAC-Net countries, there was more consumption of tetracyclines (J01A; population-consumption 2.2 versus 1.2 DDD per 1000 inhabitants per day), less consumption of other beta-lactams, predominantly cephalosporins (J01D; 2.3 versus 3.8 DDD per 1000 inhabitants per day), and less consumption of quinolone antibiotics (J01M; 1.7 versus 3.4 DDD per 1000 inhabitants per day) than in WHO Europe AMC Network countries (Supplementary Table S2). There was no or very little reported consumption of amphenicols (J01B) in ESAC-Net countries and low levels of consumption in only three countries of the WHO Europe AMC Network: Armenia (0.3 DDD per 1000 inhabitants per day, 2.2% total consumption), Kazakhstan (0.6 DDD per 1000 inhabitants per day, 3.1%) and Georgia (0.6 DDD per 1000 inhabitants per day, 2.7%).

### Trends, 2014–2018

Table 1 shows the trends in total consumption of antibacterials for systemic use (ATC group J01) for the years 2014–2018. There were statistically significant increases in total consumption over the study period in Greece (2.4%) and Iceland (4.5%). Statistically significant decreases in total consumption over the study period were reported in Denmark (CAGR −2.5%), Finland (−5.2%), Germany (−3.4%), Luxembourg (−1.3%), Netherlands (−1.5%), Norway (−2.5%), Sweden (−2.9%) and the United Kingdom (−2.6%). Only one country among the WHO Europe AMC Network countries had a statistically significant increase or decrease in total consumption over the study period, namely Bosnia and Herzegovina (+6.0%).

**TABLE 1:** Trends in total consumption of antibacterials for systemic use (ATC J01), 2014–2018.

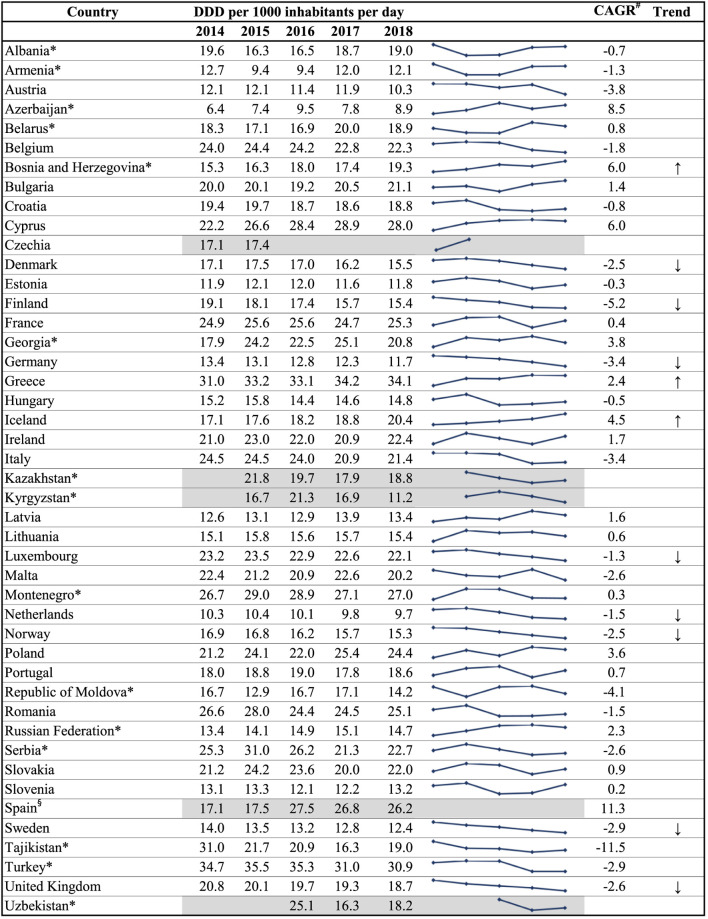

^*^ member of the WHO Europe AMC Network.

^#^ CAGR: compound average growth rate. The CAGR was only calculated where there was five years of data (2014–2018) available for the country.

^§^linear regression not applied due to changes in data sources and/or sectors for which data were reported.

↑↓arrows indicate statistically significant change.

### WHO AWaRe Classification Medicines

**FIGURE 3:**
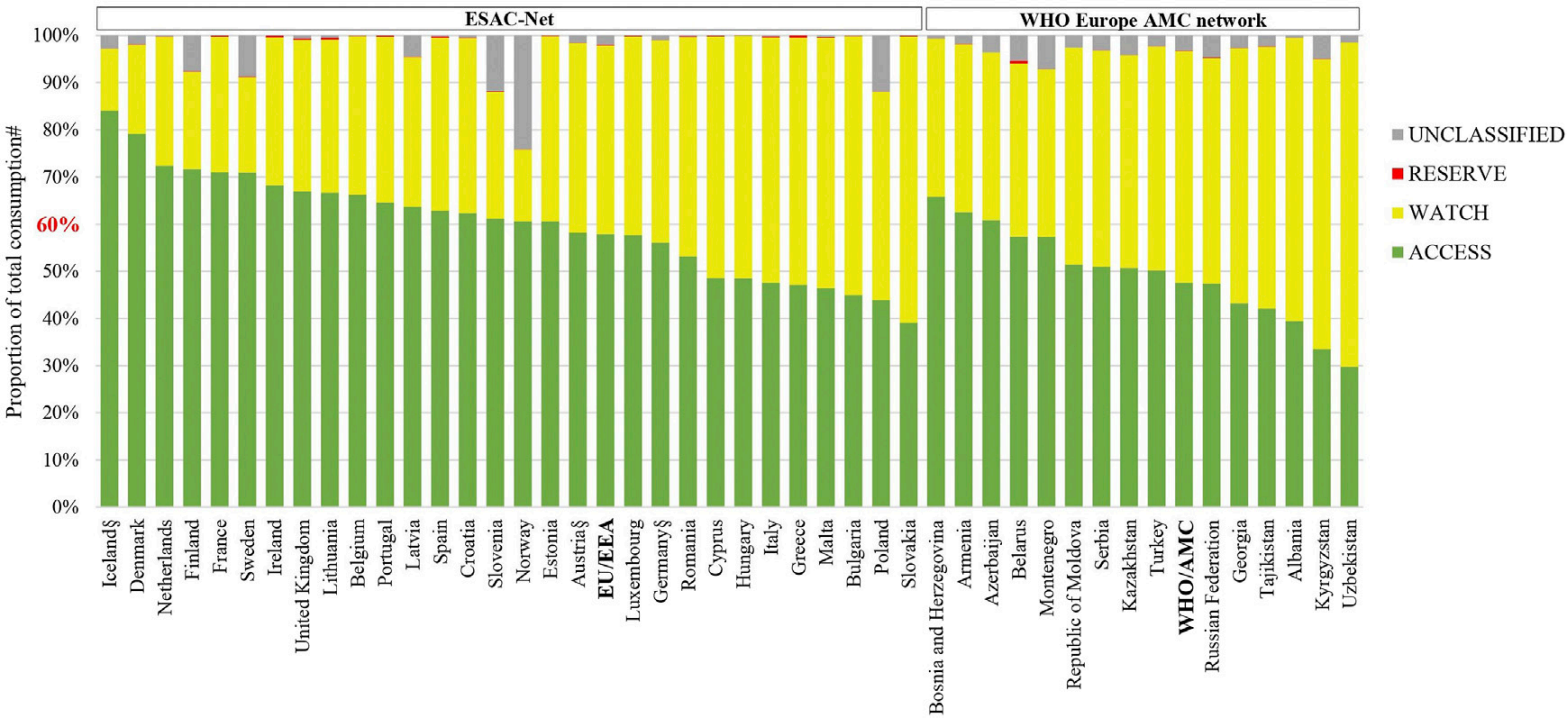
Patterns of consumption of antibacterials according to the AWaRe classification of antimicrobial agents, 2018. AWaRe: Access, Watch and Reserve classification of antimicrobials (World Health Organization 2019). EU/EEA: population-weighted mean for countries of ESAC-Net. WHO/AMC: population-weighted mean for countries of the WHO Europe AMC Network. ^#^Agents included in this analysis: antibacterials for systemic use (J01), neomycin (A07AA01), streptomycin 461 (A07AA04), polymyxin B (A07AA05), kanamycin (A07AA08), vancomycin (A07AA09), colistin 462 (A07AA10), rifamixin (A07AA11), rifampicin (J04AB02), rifamycin (J04AB03), rifabutin (J04AB04), 463 metronidazole (P01AB01). ^§^Countries for which hospital sector data were not included.

The relative consumption of AWaRe antibiotics in 2018 is shown for the two networks separately in Figure 3, and summarized in Supplementary Tables S4A and S4B. Access antibiotics comprised more than 60% of total consumption in 17 (59%) of 29 ESAC-Net countries and three (20%) of 15 WHO Europe AMC Network countries that provided 2018 data. The population-weighted mean consumption of Access agents was 57.9% for ESAC-Net and 47.4% for the WHO Europe AMC Network.

The relative consumption of Watch antibiotics ranged from 13% (Iceland) to 61% (Slovakia) of total consumption for ESAC-Net countries and from 34% (Bosnia and Herzegovina) to 69% (Uzbekistan) of total consumption for WHO Europe AMC Network countries.

Reserve antibiotics represented <1% of total consumption in all countries included in this analysis. The extent of consumption of unclassified agents varied and was the highest in Norway and Poland, representing 24 and 12% of total consumption, respectively.

Trends in the relative consumption of AWaRe agents between 2014 and 2018 are shown in Table 2. According to the WHO global indicator, i.e. 60% of total consumption should be of Access agents, 14 (48%) of 29 ESAC-Net countries achieved this target in each of the five years examined. Only one country in the WHO Europe AMC Network met this target in all five years.

**TABLE 2:** Countries achieving the target of 60% of total consumption of antibacterials being Access group agents, 2014–2018.

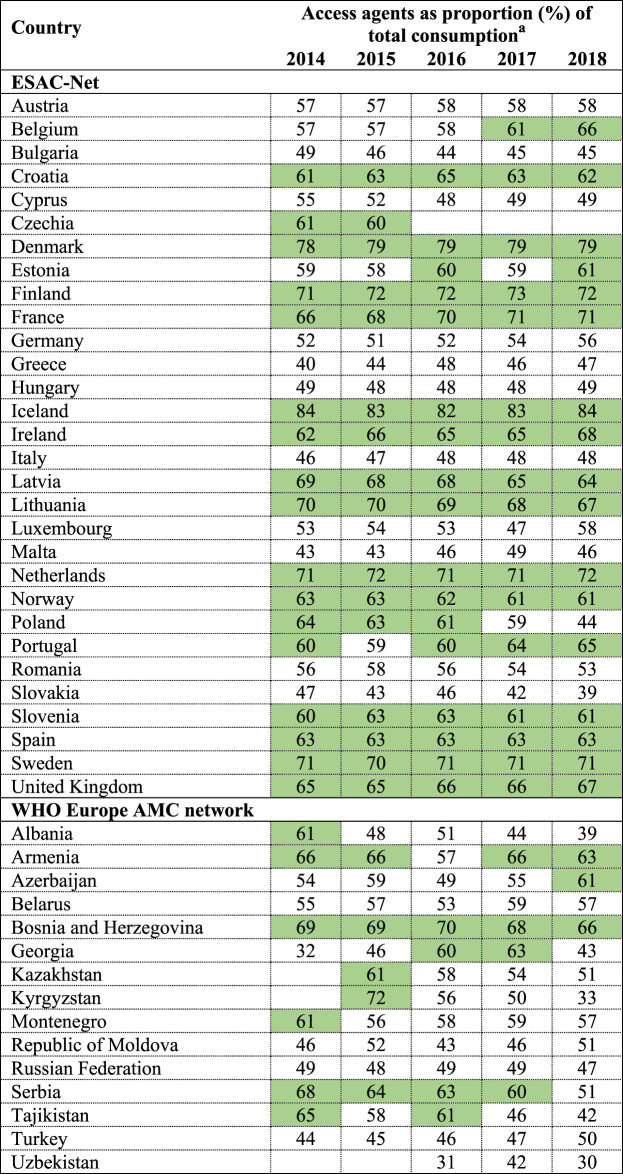

^a^ Agents included in this analysis: antibacterials for systemic use (J01), neomycin (A07AA01), streptomycin (A07AA04), polymyxin B (A07AA05), kanamycin (A07AA08), vancomycin (A07AA09), colistin (A07AA10), rifamixin (A07AA11), rifampicin (J04AB02), rifamycin (J04AB03), rifabutin (J04AB04), metronidazole (P01AB01).

Country estimates are rounded up.

Green panel identifies countries meeting the WHO global indicator that Access agents should constitute 60% of total antibacterial consumption.

### Drug Utilization 75%

Table 3 (oral agents) and Table 4 (parenteral agents) show the agents that comprise 75% of total consumption of antibacterials agents for systemic use (ATC group J01) in each of the participating countries and networks separately and summarized across each of the networks.

**TABLE 3:** Antibacterials at substance level (5th ATC group level) that compose the DU75%, oral use, 2018.

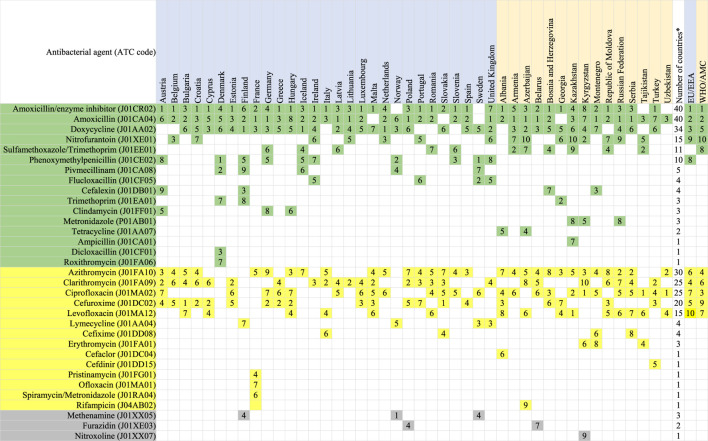

Agents included in this analysis: antibacterials for systemic use (J01), neomycin (A07AA01), streptomycin (A07AA04), polymyxin B (A07AA05), kanamycin (A07AA08), vancomycin (A07AA09), colistin (A07AA10), rifamixin (A07AA11), rifampicin (J04AB02), rifamycin (J04AB03), rifabutin (J04AB04), metronidazole (P01AB01).

The table is sorted according to the number of countries which included the antimicrobial in the DU75%, considering Access (green panel), Watch (yellow panel) and unclassified agents (gray panel) separately (World Health Organization 2019).

^*^Numbers of countries which have this agent in DU75%.

EU/EEA population-weighted mean for countries of ESAC-Net.

WHO/AMC population-weighted mean for countries of the WHO Europe AMC Network.

The numbers shown in the table refer to the frequency of use, e.g. 1 = most often consumed antimicrobial.

**TABLE 4:** Antibacterials at substance level (5th ATC group level) that compose the DU75%, parenteral use, 2018.

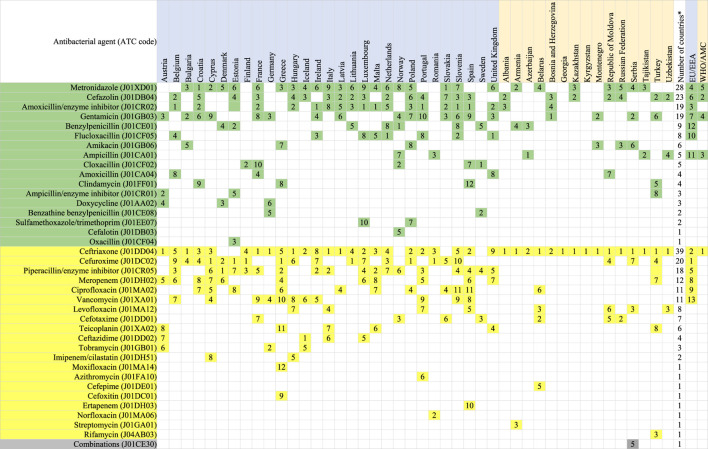

Agents included in this analysis: antibacterials for systemic use (J01), neomycin (A07AA01), streptomycin (A07AA04), polymyxin B (A07AA05), kanamycin (A07AA08), vancomycin (A07AA09), colistin (A07AA10), rifamixin (A07AA11), rifampicin (J04AB02), rifamycin (J04AB03), rifabutin (J04AB04), metronidazole (P01AB01).

The table is sorted according to the number of countries which included the antimicrobial in the DU75%, considering Access (green panel), Watch (yellow panel) and unclassified agents (gray panel) separately (World Health Organization 2019).

^*^Numbers of countries which have this agent in DU75%.

EU/EEA population-weighted mean for countries of ESAC-Net.

WHO/AMC population-weighted mean for countries of the WHO Europe AMC Network.

The numbers shown in the table refer to the frequency of use, e.g. 1 = most often consumed antimicrobial.

The number of agents constituting the DU75% for oral substances ranged from 5–9 (median 7) for ESAC-Net countries and 4–10 (median 8) for the WHO Europe AMC Network countries (Table 3). Across all countries in the network combined, the DU75% comprised 10 agents each for ESAC-Net and the WHO Europe AMC Network.

Oral amoxicillin with enzyme inhibitor (ATC code J01CR02) was the most consumed oral agent across both networks and ranked number one in 15 (52%) of 29 ESAC-Net countries and in five (33%) of 15 WHO Europe AMC Network countries. Oral amoxicillin alone (J01CA04) was ranked number two overall across both networks and ranked number one in six (21%) ESAC-Net countries and six (40%) of WHO Europe AMC Network countries.

However, there were substantial variations in the distribution of antibiotics. Three agents, phenoxymethylpenicillin (J01CE02), flucloxacillin (J01CF05) and pivmecillinam (J01CA08) were not included in the DU75% of any of the 15 WHO Europe AMC Network countries. phenoxymethylpenicillin ranked first in Denmark and Sweden, and second in Norway. Pivmecillinam ranked second in Denmark, fourth in Norway and sixth in Iceland.

Notably, Watch group agents were included in the DU75% for all countries. Across all network countries combined, Watch group agents represented four of nine agents in the DU75% for ESAC-Net and five of ten agents in the DU75% for the WHO Europe AMC Network.

Across all countries, the Watch group agents azithromycin (ATC code J01FA10), clarithromycin (J01FA09) and ciprofloxacin (J01MA02) were included in the DU75% for 30, 25 and 25 countries, respectively. However, the relative rankings of these agents varied depending on the network. In ESAC-Net, clarithromycin ranked second for consumption in six (21%) of 29 countries, third in a further four ESAC-Net countries and fourth in population-weighted estimates. Ciprofloxacin was ranked number one as most often consumed oral antibacterial in 3 (20%) of 15 WHO Europe AMC Network countries, second in another country, and third in population-weighted estimates. This agent ranked no higher than fourth in any ESAC-Net country. A second quinolone, levofloxacin (J01MA12), was the most often consumed oral antibacterial in one country of the WHO Europe AMC Network, although only ranked seventh in population-weighted estimates.

While parenteral agents represented between 2.8 and 40.1% of total consumption in 2018, there were 13 different parenteral agents included in the DU75% for ESAC-Net and five agents for the WHO Europe AMC Network. Ceftriaxone (J01DD04), a Watch group agent, ranked as the most often consumed parenteral agent in 13 (87%) of 15 WHO Europe AMC Network countries and 7 (24%) of 29 ESAC-Net countries.

## Discussion

In 2018, total consumption of antibacterials for systemic use (ATC group J01) ranged from 8.9 for Azerbaijan to 34.1 DDD per 1000 inhabitants per day for Greece, with similar ranges of estimates for ESAC-Net and the WHO Europe AMC Network and similar estimates for the population-weighted mean total consumption (19.6 and 20.0 DDD per 1000 inhabitants per day, respectively). However, there were considerable differences in the absolute and relative consumption of 3rd ATC level groups, with greater consumption of penicillins and tetracyclines, and less consumption of cephalosporins and quinolones in ESAC-Net than in WHO Europe AMC Network countries.

During 2014–2018, there were statistically significant decreases in total consumption of antibacterials for systemic use (ATC J01) in eight ESAC-Net countries: Denmark, Finland, Germany, Luxembourg, the Netherlands, Norway, Sweden and the United Kingdom. These countries all have longstanding programmes and commitments toward reducing antibiotic consumption. WHO Europe AMC Network data were characterized by more inconsistent patterns, with only one country showing a significant trend for increased consumption over 2014–2018. This may reflect the nature of the data collection, often relying on customs records and data from local manufacturers as a crude proxy for consumption in these countries, as well as shorter histories of interventions to tackle AMR. The latter is, however, starting to change among some of the WHO Europe AMC Network countries (Robertson et al., 2019) and may be reflected in future trend analyses.

The observation of similar estimates of total consumption rates in conjunction with substantially different patterns of AMC suggests that relying on indicators measuring total consumption alone is inadequate to assess national performance. More detailed analyses at the formulation, group and individual agent level are needed to identify useful targets for national interventions to improve the use of antibiotics and promote alignment of clinical practices with international guidance on their responsible use. This is particularly important if patterns of infectious diseases and AMR, as well as other determinants of AMC including differences in patient expectations, vary between countries (Fletcher-Lartey et al., 2016; Sirota et al., 2017; and; Ritchie et al., 2019). Several disparities reflect the fact that certain antibiotics, for example, phenoxymethylpenicillin, flucloxacillin and pivmecillinam, are not registered in some countries. Other disparities, such as more reliance on parenteral products and greater use of quinolones in WHO Europe AMC Network than ESAC-Net countries, are more likely to reflect prescribing practices and cultural preferences and are potential targets for interventions and behavior change.

High levels of consumption of Watch agents are an obvious target for interventions including review of clinical guidelines and prescribing algorithms. Low levels of consumption of Reserve agents in this study reflect the analysis of data on total consumption combining community and hospital sectors and different consumption patterns would be expected in analyses of hospital data alone. While some classes of agents will be used almost exclusively in the hospital setting, on average, 90% of overall national antibiotic consumption across European countries is community consumption. Disaggregation of data to hospital and community sectors was not possible in most WHO Europe AMC Network countries; however, this is an important future development as countries strengthen and enhance their surveillance capacity and recognize the value of disaggregated data to inform stewardship activities in the community and hospital sectors.

Fourteen ESAC-Net countries met WHO’s suggested national target of 60% of total consumption of antibacterials being derived from the Access list in each of the five years analyzed. This suggests that more ambitious targets may be warranted in high-income countries that already have implemented comprehensive monitoring and intervention programmes to address AMR. In contrast, only one country in the WHO Europe AMC Network achieved this target in each of the five years. The findings in the high-income countries compare well with Canada where there was evidence of increasing relative consumption of Access agents between 2014 and 2018, reaching 67.5% of total consumption in 2018 (Public Health Agency of Canada, 2020).

Despite the expansion of the AWaRe classification in 2019 to include 180 antibacterials used globally (World Health Organization, 2019), three oral agents, methenamine (J01XX05), furazidin (J01XE03) and nitroxoline (J01XX07), all used for the prophylaxis or treatment of urinary tract infections, yet remain unclassified, but appear in the DU75% for four ESAC-Net countries and two WHO Europe AMC Network countries. There is an opportunity for WHO in the future to address the limitations in the current list and to expand the AWaRe classification to include antimicrobials that are commonly used in some countries. Unclassifed agents constituted 24.1% of total consumption in Norway in 2018, mostly accounted for by methenamine, which is commonly used for prophylaxis of urinary tract infections in elderly women and is not considered to be an AMR-inducing agent (Alberg et al., 2017). Our estimates of the consumption of unclassified agents differ from those reported by Klein et al., (2020). These authors used expert consultations to classify 54 of 233 antibiotics included in the sales data of the IQVIA database that are currently not included in the AWaRe classification, leaving only three agents in their unclassified group.

As noted in other studies, the number of antimicrobials constituting the DU75% by substance was small (Zarb et al., 2011) with a median of seven and eight different oral agents in ESAC-Net and WHO Europe AMC Network countries, respectively. Linking the DU75% analysis to the AWaRe classification adds further value, illustrating differences in choices of agents between countries and networks, their ranking by consumption rates and identifying potential targets for interventions to improve practices.

The limitations of some of the data have implications for interpretation of results. Only medicines with an assigned ATC code and DDD are included in the analyses. Where there are medicines without codes consumed by the population, estimates of DDD per 1000 inhabitants per day will be underestimated. While import records have limitations, they will include over-the-counter supply of antibacterials without prescription that occurs in some countries. Differences in data sources can limit some comparisons. Supply cycles may influence consumption estimates in some settings while customs records and data from local manufacturers are a crude proxy for consumption. However, in the absence of more robust data, using these sources allow countries to start the important task of monitoring antibacterial consumption as part of national efforts to address AMR. The ATC/DDD methodology provides a common technical basis for surveillance of antimicrobial consumption. Where sources remain the same, it is possible to discern trends more reliably in volumes and patterns of consumption over time.

Despite similar estimates of total consumption of antibacterials for systemic use (ATC J01) in the Netherlands and in Azerbaijan, 9.7 and 8.9 DDD per 1000 inhabitants per day, respectively, the situation in the two countries is different. Trend analyses demonstrate statistically significant reductions in total consumption in the Netherlands but increasing total consumption in Azerbaijan during 2014–2018. The low starting baseline of consumption estimates for Azerbaijan in 2014 and variability over the study period could suggest poor access to antibiotics, incomplete capture of import and local manufacturing information, the impact of medicines import cycles, or combinations of these factors. Nevertheless, this increase in total consumption over time is consistent with the findings of other studies linking increasing consumption in low- and middle-income countries to growth in gross domestic product per capita (Klein et al., 2018), and the quality of governance in the pharmaceutical sector including regulatory practices and enforcement of regulations (Rönnerstrand and Lapuente, 2017). Malik and Bhattacharyya (2019) modeled complex associations between socio-economic growth, ecology of infectious disease and antibiotic misuse, particularly through self-medication, suggesting there are synergistic interactions of these components feeding back on each other and contributing to AMR.

Similar to the large variations in antibiotic consumption between countries described by this study and other global analyses (Klein et al., 2020), considerable differences in the proportion of AMR in key pathogens have been reported for both EU/EEA countries (European Centre for Disease Prevention and Control, 2019b) and non-EU/EEA European countries (World Health Organization Regional Office for Europe, 2018). Associations between national antimicrobial consumption rates and national AMR levels, in both the hospital and community sector, have been reported for EU/EEA countries (European Centre for Disease Prevention and Control and European Food Safety Authority and European Medicines Agency, 2017b), highlighting the importance of addressing antibiotic consumption as part of national efforts towards reducing the burden of AMR in Europe (Wojkowska-Mach et al., 2018). Furthermore, disaggregation of data to community and hospital sectors facilitates monitoring using sector-specific metrics for quantifying antibiotic use and assessing the quality of prescribing (Coenen et al., 2007; de Bie et al., 2016; Stanic Benic et al., 2018). Hospital antimicrobial stewardship programmes that generally focus on changes to antimicrobial use practices have also demonstrated their value in delivering clinical and economic benefits, with reductions in length of stay a key driver of cost savings (Nathwani et al., 2019). Community interventions can be developed, building on existing evidence that communication skills training and changing patient expectations to receive antibiotics can lead to significant reductions in antibiotic prescriptions for viral upper respiratory tract infections (Ritchie et al., 2019; Strumann et al., 2020).

In conclusion, the development and implementation of effective national policies to deal with AMR vary across countries in both ESAC-Net and the WHO Europe AMC Network. Reliable data are needed to describe patterns of AMC and to monitor the evolution of AMR. Standardized methods of data collection and analysis and the application of common metrics and indicators facilitate benchmarking across settings, countries and regions. The quantitative estimates presented should be considered a starting point for further studies to understand better the use of these medicines in clinical practice – this will require further quantitative and qualitative studies in primary care and hospital sectors at the country level.

The longer history and experience of ESAC-Net provides an important platform from which to build capacity for monitoring and evaluation in the WHO Europe AMC Network. Subsequent cross-national comparisons, especially including quality measures, can stimulate national and regional discussions and promote activities and interventions to address problem practices. Timely data are important if they are to be used to guide policy developments at the national level. This study included data for 2018 and captured information on patterns of antibiotic consumption in countries in Eastern Europe and Central Asia that are currently not well represented in global sales databases such as IQVIA (Klein et al., 2020).

Several factors support increased engagement and information exchange between ESAC-Net and the WHO Europe AMC Network. Beyond geographic proximity, several members of the WHO Europe AMC Network are candidate countries for accession to the EU (European Commission, 2020b). In advance of membership, these countries must revise their national legislation to be able to apply the EU’s acquis, the body of common rights and obligations that are binding on all EU Member States. This will promote harmonization of procedures for registration of pharmaceutical products including antibiotics across Europe. Furthermore, strategic partnerships are a cornerstone of the 2020–2025 WHO European Programme of Work (World Health Organization Regional Office for Europe 2020c), and this will facilitate building on complementary work already being undertaken by agencies aligned with the European Commission and WHO Europe. Overall, we believe that this study illustrates the value of harmonized approaches to data collection and analysis of antibiotic consumption data that can underpin Europe-wide strategies to address AMR. In parallel with joint reporting of AMR data from the European Antimicrobial Resistance Surveillance Network (EARS-Net) and the WHO Europe Central Asian and European Surveillance of Antimicrobial Resistance (CAESAR) network, we plan further reporting on joint ESAC-Net and WHO Europe AMC surveillance in the future.

### Group Authors

Authors contributing to the ESAC-Net and WHO Europe AMC Network study groups are shown below.

**Table:** 

Authors contributing to the ESAC-Net study group:		
Austria	Reinhild Strauss	Federal Ministry for Social Affairs, Health, Care and Consumer Protection
Belgium	Eline Vandael	Sciensano
Bulgaria	Ivan N. Ivanov	National Center of Infectious and Parasitic Diseases
Croatia	Marina Payerl-Pal	The Interdisciplinary Section for Antibiotic Resistance Control, Ministry of Health
Cyprus	Isavella Kyriakidou	Pharmaceutical Services, Ministry of Health
Denmark	Majda Attauabi	Statens Serum Institut
Estonia	Elviira Linask	Estonian State Agency of Medicines
France	KarimaHider - Mlynarz	EPI-PHARE, epidemiology of health products (French National Agency for the Safety of Medicines and Health Products, and French National Health Insurance)
Germany	Birgitta Schweickert	Robert Koch Institute, Department: Healthcare-associated Infections, Surveillance of Antimicrobial Resistance and Consumption, Berlin
Hungary	Ria Benkö	University of Szeged, Department of Clinical Pharmacy, Albert Szent Györgyi Health Center, Central Pharmacy and Department of Emergency Medicine
Iceland	Guðrún Aspelund	Center for Health Security and Communicable Disease Control, Directorate of Health, Reykjavik
Ireland	Karen Burns	Health Protection Surveillance Center, Dublin
Italy	Filomena Fortinguerra	Italian Medicines Agency (AIFA), Rome
Latvia	Ieva Rutkovska	Centre for Disease Prevention and Control, Department of Infectious Disease Surveillance and Immunization, Riga
Luxembourg	Martine Trauffler	Division of Pharmacy and Medicines, Directorate of Health
Malta	Peter Zarb	National Antibiotic Committee
Netherlands	Stephanie Natsch	Department of Pharmacy, Radboud university medical center, Nijmegen
Norway	Hege Salvesen Blix	Norwegian Institute of Public Health
Poland	Anna Olczak-Pieńkowska	National Medicines Institute, Department of Epidemiology and Clinical Microbiology
Portugal	Ana Silva	INFARMED - National Authority of Medicines and Health Products, I.P.
Romania	Ionel Iosif	National Institute of Public Health, National Center for Disease Prevention and Control, Bucharest
Slovakia	Tomáš Tesař	Department of Organization and Management in Pharmacy, Pharmaceutical Faculty, Comenius University in Bratislava
Slovenia	Milan Cižman	University Medical Center, Department of Infectious Diseases, Ljubljana
Spain	Mayte Alonso	National Agency for Drugs and Medical Devices
Sweden	Vendela Bergfeldt	Public Health Agency of Sweden
United Kingdom	Susan Hopkins	National Infection Service, Public Health England

Authors contributing to the WHO Europe AMC Network study group:		
Albania	Narvina Sinani	National Agency for Drugs and Medical Devices
Armenia	Lilit Ghazaryan	Scientific Center of Drug and Medical Technology Expertize of Ministry of Health
Azerbaijan	Fikriyya Ibrahimli	Analytical Expertize Center of Ministry of Health
Bosnia and Herzegovina	Tijana Spasojević	Sector for Providing Information on Drugs and Medical Products in Agency for Medicinal Products and Medical Devices of Bosnia and Herzegovina
Belarus	Halina Pyshnik	Department of Organization of Medicines Supply, Ministry of Health
Georgia	Marine Baidauri	Health Policy Division of Policy Department Ministry of IDPs, Labor, Health and Social Affairs of Georgia
Kazakhstan	Larissa Makalkina	Department of Clinical Pharmacology, Astana Medical University Republican Public Union "Professional Association of Clinical Pharmacologists and Pharmacists"
Kyrgyzstan	Aizhan Tabaldyeva	Unit on Specialized Expertize of Medicines of Department of Drug Provision and Medical Devices, Ministry of Health
Republic of Moldova	Angela Carp	P.I. Coordination, Implementation and Monitoring Unit of the Health System Projects
Montenegro	Lidija Čizmović	Department for Establishing Maximum Prices and Monitoring Consumption of Medicines, Agency for Medicines and Medical Devices
Russian Federation	Svetlana Rachina	Internal Medicine Department №2, I.M. Sechenov First Moscow State Medical University, Moscow, Russian Federation
Serbia	Milica Bajčetić	Department of Pharmacology, Clinical Pharmacology and Toxicology, Faculty of Medicine, University of Belgrade, Serbia
Tajikistan	Nargis Kalandarova	Department of Conformity Assessment of Medicines of the State Unitary Enterprise for Examination and Testing Pharmaceutical and Medical Products of the State Service Health and Social Protection of Population Supervision
Turkey	Mesil Aksoy	Department of Rational Use of Medicines. Turkish Medicines and Medical Devices Agency, Ministry of Health of Turkey
Uzbekistan	Mukhabbat Ibragimova	Agency on Development of the Pharmaceutical Industry under the Ministry of Health of the Republic of Uzbekistan. Head of Information and Analytics Department

## Data Availability

The data analyzed in this study are subject to the following licenses/restrictions: Data from ESAC-Net are publicly accessible from the ESAC-Net interactive database and under some circumstances after submission of a request for access to data to The European Surveillance System. The European Surveillance System database allows for correction and re-uploading of historical data by the reporting countries. Therefore, the results presented here may differ from the most recent available data at the public interactive ESAC-Net database. No further data are available for the WHO Europe AMC Network. Requests to access ESAC-Net datasets should be directed to https://www.ecdc.europa.eu/en/publications-data/european-surveillance-system-tessy.
