# Corrigendum: Variations in the Consumption of Antimicrobial Medicines in the European Region, 2014–2018: Findings and Implications From ESAC-Net and WHO Europe

**DOI:** 10.3389/fphar.2021.765748

**Published:** 2021-09-30

**Authors:** Jane Robertson, Vera Vlahović-Palčevski, Kotoji Iwamoto, Liselotte Diaz Högberg, Brian Godman, Dominique L. Monnet, Sarah Garner, Klaus Weist, ESAC-Net Study Group and WHO Europe AMC Network Study Group

**Affiliations:** ^1^ World Health Organization Regional Office for Europe, Copenhagen, Denmark; ^2^ Department of Clinical Pharmacology, The University of Newcastle, Callaghan, NSW, Australia; ^3^ Department of Clinical Pharmacology, University Hospital Rijeka/Medical Faculty and Faculty of Health Studies, University of Rijeka, Rijeka, Croatia; ^4^ European Centre for Disease Prevention and Control, Solna, Sweden; ^5^ Strathclyde Institute of Pharmacy and Biomedical Sciences, University of Strathclyde, Glasgow, United Kingdom; ^6^ Department of Public Health Pharmacy and Management, School of Pharmacy, Sefako Makgatho Health Sciences University, Pretoria, South Africa

**Keywords:** antibiotic utilization, antimicrobial medicines consumption, AWaRe classification, cross-national comparative study, drug utilization 75%, European Surveillance of Antibiotic Consumption Network, Eastern Europe, Central Asia

In the Discussion, subsection Group Authors, a name was incorrectly spelled as Leva Rutkovska. The correct spelling is Ieva Rutkovska.

The authors apologize for this error and state that this does not change the scientific conclusions of the article in any way. The original article has been updated.

