# Supplementary material for: Variations in the Consumption of Antimicrobial Medicines in the European Region, 2014–2018: Findings and Implications from ESAC-Net and WHO Europe
Source: Front Pharmacol. 2021 Jun 17;12:639207. doi: 10.3389/fphar.2021.639207 (PMC8248674; doi:10.3389/fphar.2021.639207)
Supplement: Supplementary file 1 [file DataSheet1.docx]

Supplementary Material

**Table 1A. Sources of data used for calculating antibiotic consumption, ESAC-Net, 2014-2018**

| **Country** | **2014** | | **2015** | | **2016** | | **2017** | | **2018** | |
| --- | --- | --- | --- | --- | --- | --- | --- | --- | --- | --- |
|  | **AC** | **HC** | **AC** | **HC** | **AC** | **HC** | **AC** | **HC** | **AC** | **HC** |
| Austria | R |  | R |  | R |  | R |  | R |  |
| Belgium | R | R | R | R | R | R | R | R | R | R |
| Bulgaria | S | S | S | S | S | S | S | S | S | S |
| Croatia | R | R | R | R | R | R | R | R | R | R |
| Cyprus* | S |  | S |  | S |  | S |  | S |  |
| Czechia | R |  | R |  |  |  |  |  |  |  |
| Denmark | S | S | S | S | S | S | S | S | S | S |
| Estonia | S | S | S | S | S | S | S | S | S | S |
| Finland | S | S | S | S | S | S | S | S | S | S |
| France | S | S | S | S | S | S | S | S | S | S |
| Germany§ | R |  | R |  | R |  | R |  | R |  |
| Greece | S | S | S | S | S | S | S | S | S | S |
| Hungary | S | S | S | S | S | S | S | S | S | S |
| Iceland | S |  | S |  | S |  | S |  | S |  |
| Ireland | S | B | S | B | S | B | S | B | S | B |
| Italy | B | R | B | R | B | R | B | R | B | R |
| Latvia | S | S | S | S | S | S | S | S | S | S |
| Liechtenstein |  |  |  |  |  |  |  |  |  |  |
| Lithuania | S | S | S | S | S | S | S | S | S | S |
| Luxembourg§ | R | R | R | S | R | S | R | S | R | S |
| Malta | S | S | S | S |  |  |  |  |  |  |
| Netherlands§ | S | S | S | S | S | S | S | S | S | S |
| Norway | B | B | B | B | B | B | B | B | B | B |
| Poland | S | S | S | S | S | S | S | S | S | S |
| Portugal | R | B | R | B | R | B | R | B | R | B |
| Romania* | S |  | S |  | S |  | S |  | B |  |
| Slovakia | S |  | S |  | S |  | S | S | S | S |
| Slovenia | B | R | B | R | B | R | B | R | B | R |
| Spain | R |  | R |  | B | S | B | S | B | S |
| Sweden | S | S | S | S | S | S | S | S | S | S |
| United Kingdom | R | B | B | B | R | R | R | R | R | B |

AC: community (primary care) sector; HC: hospital sector

S: sales data; R: reimbursement data; B: both sales data and reimbursement data. Reimbursement data do not include consumption of antimicrobials obtained without prescription and other non-reimbursed courses compared to sales data

* Country only provided total care data, i.e. community and hospital sector data combined, in 2018

§ coverage of reimbursement data in Germany is 85%, in the Netherlands 92% and Luxembourg 91%.

**Table 1B. Sources of data used for calculating antibiotic consumption, WHO Europe AMC Network, 2014-2018**

| **Country** | **2014** | **2015** | **2016** | **2017** | **2018** |
| --- | --- | --- | --- | --- | --- |
| Albania | I | I | I | I | I |
| Armenia | I, S | I, S | I, S | I, S | I, S |
| Azerbaijan | I | I | I | I | I |
| Bosnia and Herzegovina | S | S | S | S | S |
| Belarus | I, M | I, M | I, M | I, M | I, M |
| Georgia | I | I | I | I | I |
| Kazakhstan |  | S | S | S | S |
| Kyrgyzstan§ |  | I, S | I, S | I, S | I, S |
| Montenegro | S | S | S | S | S |
| Republic of Moldova | I, M | I, M | I, M | I, M | I, M |
| Russian Federation | S | S | S | S | S |
| Serbia | S | S | S | S | S |
| Tajikistan | I, C | I, C | I, C | I, C | I, C |
| Turkey# | S | S | S | S | S |
| Uzbekistan |  |  | I, S | I, S | I, S |

S: sales data; I: import records; M: manufacturing records; C: certification records

§ coverage of sales data in Kazakhstan is 80%

# Turkey uses wholesalers records from pharmaceutical track and trace system.

**Table 2. Total consumption of antibacterials for systemic use use (ATC J01) expressed in DDD per 1000 per day, by pharmacological subgroup, 2018**

| **Country** | **Beta-lactam antibacterials, penicillins (J01C)** | **Other beta-lactam antibacterials (J01D)** | **Tetracyclines (J01A)** | **Macrolides, lincosamides and streptogramins (J01F)** | **Quinolone antibacterials (J01M)** | **Sulfonamides and trimethoprim (J01E)** | **Amphenicols (J01B)** | **Other J01 antibacterials (J01G, J01R, J01X)** | **Total** |
| --- | --- | --- | --- | --- | --- | --- | --- | --- | --- |
|  | **DDD per 1000 inhabitants per day (% of total)** | | | | | | | | |
| Greece | 11.4 (33.4) | 8.4 (24.7) | 3.0 (8.8) | 6.5 (19.1) | 3.2 (9.3) | 0.3 (0.9) | <0.1  (<0.1) | 1.3 (3.8) | 34.1 |
| Turkey* | 12.4 (40.2) | 8.0  (26.0) | 1.3 (4.1) | 4.4 (14.1) | 3.0 (9.7) | 0.3 (0.9) | - | 1.5 (5.0) | 30.9 |
| Cyprus | 8.9 (31.7) | 5.9 (21.0) | 3.8 (13.6) | 3 (10.9) | 5.3 (18.9) | 0.3 (0.9) | - | 0.8 (2.9) | 28.0 |
| Montenegro* | 9.9 (36.5) | 5.4 (20.1) | 1.3 (4.8) | 4.8 (17.9) | 3.3 (12.0) | 0.8 (2.9) | - | 1.5 (5.7) | 27.0 |
| Spain | 14.5 (55.1) | 2.8 (10.5) | 1.5 (5.7) | 3.2 (12.2) | 3.0 (11.4) | 0.5 (1.8) | - | 0.9 (3.3) | 26.2 |
| France | 14.2 (56.0) | 1.7 (6.8) | 3.2 (12.5) | 3.0 (11.9) | 1.5 (6.0) | 0.5 (1.8) | <0.1  (<0.1) | 1.2 (4.9) | 25.3 |
| Romania | 11.4 (45.3) | 5.2 (20.6) | 0.9 (3.8) | 3.0 (12.0) | 3.3 (13.1) | 0.8 (3.3) | <0.1  (<0.1) | 0.5 (1.8) | 25.1 |
| Poland | 7.1 (29.0) | 3.5 (14.2) | 2.3 (9.6) | 6.1 (25.0) | 1.7 (6.8) | 0.6 (2.4) | - | 3.2 (13.0) | 24.4 |
| Serbia* | 6.8 (29.9) | 3.6 (15.7) | 1.7 (7.3) | 5.3 (23.3) | 4.0 (17.6) | 0.7 (3.1) | - | 0.7 (3.2) | 22.7 |
| Ireland | 10.6 (47.1) | 1.3 (6.0) | 2.7 (11.9) | 4.3 (19.1) | 0.9 (3.9) | 1.1 (4.8) | - | 1.6 (7.3) | 22.4 |
| Belgium | 10.9 (48.7) | 1.6 (7.3) | 1.9 (8.5) | 3.7 (16.5) | 1.3 (6.0) | 0.2 (1.1) | <0.1  (<0.1) | 2.6 (11.9) | 22.3 |
| Luxembourg | 9.0 (40.8) | 3.3 (14.9) | 2.1 (9.4) | 3.6 (16.2) | 2.2 (10.0) | 0.3 (1.4) | <0.1  (<0.1) | 1.6 (7.2) | 22.1 |
| Slovakia | 5.3 (24.3) | 6.7 (30.5) | 1.7 (7.9) | 4.9 (22.1) | 2.3 (10.7) | 0.4 (1.8) | <0.1  (<0.1) | 0.6 (2.7) | 22.0 |
| Italy | 9.5 (44.4) | 2.5 (11.7) | 0.5 (2.6) | 4.1 (19.0) | 3.0 (14.1) | 0.9 (4.0) | 0.1 (0.2) | 0.9 (4.1) | 21.4 |
| Bulgaria | 5.6 (26.7) | 5.4 (25.5) | 1.6 (7.7) | 4.1 (19.5) | 3.0 (14.2) | 0.9 (4.1) | <0.1 (0.2) | 0.4 (2.1) | 21.1 |
| Georgia* | 4.3 (20.6) | 7.2 (34.6) | 1.1 (5.3) | 1.9 (9.1) | 2.0 (9.6) | 2.2 (10.5) | 0.6 (2.7) | 1.6 (7.5) | 20.8 |
| Iceland | 8.9 (43.7) | 0.6 (2.8) | 5.0 (24.6) | 1.6 (7.7) | 0.8 (4.0) | 2.5 (12.0) | - | 1.1 (5.2) | 20.4 |
| Malta | 6.9 (34.4) | 2.9 (14.6) | 1.6 (8.0) | 4.6 (22.9) | 2.6 (12.9) | 0.4 (1.8) | - | 1.1 (5.4) | 20.2 |
| **EU/EEA** | 8.7 (43.8) | 2.3 (11.5) | 2.2 (11.1) | 3.2 (15.9) | 1.7 (8.5) | 0.6 (3.1) | - | 1.2 (6.2) | 20.0 |
| **WHO/AMC** | 6.3 (31.9) | 3.8 (19.3) | 1.2 (6.1) | 3.0 (15.4) | 3.4 (17.1) | 0.5 (2.5) | 0.1 (0.6) | 1.4 (6.9) | 19.6 |
| Bosnia and Herzegovina* | 7.6 (39.4) | 3.0 (15.4) | 1.2 (6.3) | 2.4 (12.2) | 2.5 (13.1) | 1.8 (9.5) | - | 0.8 (4.0) | 19.3 |
| Tajikistan* | 2.7 (14.2) | 3.4 (17.9) | 0.7 (3.8) | 1.4 (7.6) | 6.1 (32.1) | 2.5 (13.1) | 0.1 (0.5) | 2.1 (10.8) | 19.0 |
| Albania* | 4.8 (25.0) | 6.0 (31.4) | 2.0 (10.6) | 2.2 (11.8) | 3.5 (18.4) | 0.3 (1.6) | <0.1 | 0.2 (1.1) | 19.0 |
| Belarus* | 7.1 (37.6) | 2.2 (11.7) | 2.3 (12.4) | 2.9 (15.5) | 2.0 (10.6) | 0.2 (0.9) | 0.1 (0.4) | 2.0 (10.8) | 18.9 |
| Kazakhstan* | 4.5 (23.9) | 3.1 (16.2) | 1.3 (6.7) | 2.1 (11.4) | 4.8 (25.5) | 0.7 (3.7) | 0.6 (3.1) | 1.8 (9.5) | 18.8 |
| Croatia | 8.3 (44.2) | 3.1 (16.2) | 1.0 (5.4) | 3.0 (15.9) | 1.7 (9.2) | 0.5 (2.8) | - | 1.2 (6.2) | 18.8 |
| United Kingdom | 7.3 (39.2) | 0.4 (2.0) | 4.9 (26.1) | 2.9 (15.8) | 0.6 (3.0) | 1.0 (5.2) | <0.1  (<0.1) | 1.6 (8.6) | 18.7 |
| Portugal | 9.7 (52.3) | 2.0 (10.8) | 0.9 (4.7) | 2.8 (15.3) | 1.3 (7.3) | 0.4 (2.2) | - | 1.4 (7.3) | 18.6 |
| Uzbekistan* | 3.9 (21.4) | 3.2 (17.7) | 0.4 (2.4) | 3.3 (18) | 6.7 (36.7) | - | 0.1 (0.8) | 0.6 (3.1) | 18.2 |
| Denmark | 10.2 (65.9) | 0.3 (1.8) | 1.4 (9.3) | 1.6 (10.4) | 0.5 (3.5) | 0.8 (5.1) | - | 0.6 (4.0) | 15.5 |
| Finland | 5.0 (32.1) | 2.9 (18.8) | 3.2 (20.8) | 0.8 (5.4) | 0.8 (5.3) | 1.1 (6.9) | - | 1.6 (10.7) | 15.4 |
| Lithuania | 7.2 (46.6) | 2.1 (13.7) | 1.4 (9.0) | 2.2 (14.2) | 1.1 (6.9) | <0.1 (0.1) | - | 1.5 (9.7) | 15.4 |
| Norway | 6.1 (39.9) | 0.3 (2.1) | 2.7 (17.7) | 1.0 (6.2) | 0.4 (2.4) | 0.7 (4.9) | <0.1  (<0.1) | 4.1 (26.9) | 15.3 |
| Hungary | 4.9 (33.0) | 2.5 (16.8) | 1.1 (7.7) | 3.0 (19.9) | 2.5 (16.9) | 0.5 (3.2) | - | 0.4 (2.5) | 14.8 |
| Russian Federation* | 4.1 (27.9) | 1.8 (12.4) | 1.3 (8.7) | 2.6 (17.5) | 2.9 (19.6) | 0.5 (3.4) | 0.1 (1.0) | 1.4 (9.5) | 14.7 |
| Republic of Moldova* | 4.2 (29.9) | 3.0 (21.2) | 0.6 (4.2) | 2.0 (13.9) | 2.0 (14.4) | 1.1 (7.5) | <0.1 (0.2) | 1.2 (8.7) | 14.2 |
| Latvia | 4.8 (36.0) | 1.2 (9.0) | 2.2 (16.4) | 2.2 (16.3) | 1.2 (9.0) | 0.8 (6.1) | - | 1 (7.3) | 13.4 |
| Slovenia | 7.5 (57.1) | 0.6 (4.8) | 0.5 (4.0) | 1.9 (14.5) | 1.3 (9.9) | 0.7 (5.7) | - | 0.5 (4.0) | 13.2 |
| Sweden | 6.6 (53.5) | 0.2 (1.9) | 2.3 (18.7) | 0.6 (4.5) | 0.7 (6.0) | 0.3 (2.5) | - | 1.6 (12.8) | 12.4 |
| Armenia* | 3.4 (27.9) | 1.1 (8.9) | 1.6 (13.6) | 1.7 (13.9) | 1.7 (14.5) | 1.4 (11.2) | 0.3 (2.2) | 0.9 (7.7) | 12.1 |
| Estonia | 4.3 (36.3) | 1.7 (14.3) | 1.3 (10.9) | 2.5 (21.2) | 0.9 (7.3) | 0.5 (4.1) | - | 0.7 (5.9) | 11.8 |
| Germany | 3.7 (32.1) | 2.5 (21.3) | 1.6 (14.0) | 1.9 (16.7) | 1.0 (8.2) | 0.4 (3.7) | - | 0.5 (4.0) | 11.7 |
| Kyrgyzstan* | 1.6 (14.0) | 3.9 (35.1) | 0.6 (5.0) | 1.4 (12.6) | 1.8 (16.5) | <0.1 (0.3) | 0.1 (1.2) | 1.7 (15.3) | 11.2 |
| Austria | 4.7 (45.7) | 1.4 (13.6) | 0.4 (3.5) | 2.3 (21.8) | 1.0 (10.1) | 0.2 (2.0) | - | 0.3 (3.3) | 10.3 |
| Netherlands | 3.2 (32.7) | 0.3 (2.6) | 2.0 (20.3) | 1.5 (15.5) | 0.8 (8.4) | 0.5 (4.7) | - | 1.5 (15.7) | 9.7 |
| Azerbaijan* | 3.0 (33.4) | 0.7 (7.5) | 1.5 (16.8) | 1.4 (15.6) | 0.9 (9.8) | 0.6 (6.6) | 0.1 (0.8) | 0.8 (9.5) | 8.9 |

*WHO Europe AMC Network

**Table 3. Total consumption of antibacterials for systemic use (ATC J01) in DDD per 1000 inhabitants per day, by route of administration, 2018**

| **Country** | **Oral** | | **Parenteral** | | **Total** |
| --- | --- | --- | --- | --- | --- |
|  | **DDD per 1000 inhabitants per day** | **%** | **DDD per 1000 inhabitants per day** | **%** | **DDD per 1000 inhabitants per day** |
| Greece | 32.1 | 94.1 | 2.0 | 5.9 | 34.1 |
| Turkey* | 30.0 | 97.2 | 0.9 | 2.8 | 30.9 |
| Cyprus | 26.7 | 95.4 | 1.3 | 4.6 | 28.0 |
| Republic of Moldova* | 24.8 | 91.6 | 2.3 | 8.4 | 27.0 |
| Spain | 25.2 | 96.2 | 1.0 | 3.8 | 26.2 |
| France | 24.4 | 96.4 | 0.9 | 3.6 | 25.3 |
| Romania | 19.1 | 76.1 | 6.0 | 23.9 | 25.1 |
| Poland | 23.4 | 96.0 | 1.0 | 4.0 | 24.4 |
| Serbia* | 21.2 | 93.4 | 1.5 | 6.6 | 22.7 |
| Ireland | 21.3 | 95.0 | 1.1 | 5.0 | 22.4 |
| Belgium | 21.2 | 95.4 | 1.0 | 4.6 | 22.3 |
| Luxembourg | 21.1 | 95.7 | 1.0 | 4.3 | 22.1 |
| Slovakia | 20.8 | 94.8 | 1.1 | 5.2 | 22.0 |
| Italy | 20.0 | 93.7 | 1.3 | 6.3 | 21.4 |
| Bulgaria | 19.5 | 92.5 | 1.6 | 7.5 | 21.1 |
| Georgia* | 14.3 | 68.4 | 6.6 | 31.6 | 20.8 |
| Iceland§ | 20.4 | 100.0 | 0.0 | 0.0 | 20.4 |
| Malta | 18.8 | 93.2 | 1.4 | 6.8 | 20.2 |
| Belarus* | 18.3 | 94.9 | 1.0 | 5.1 | 19.3 |
| Tajikistan* | 13.9 | 73.0 | 5.1 | 27.0 | 19.0 |
| Albania* | 17.5 | 92.2 | 1.5 | 7.8 | 19.0 |
| Bosnia and Herzegovina* | 16.7 | 88.5 | 2.2 | 11.5 | 18.9 |
| Kazakhstan* | 15.3 | 81.4 | 3.5 | 18.6 | 18.8 |
| Croatia | 17.7 | 94.1 | 1.1 | 5.9 | 18.8 |
| United Kingdom | 17.7 | 94.5 | 1.0 | 5.5 | 18.7 |
| Portugal | 17.5 | 94.3 | 1.1 | 5.7 | 18.6 |
| Uzbekistan* | 14.0 | 76.7 | 4.2 | 23.3 | 18.2 |
| Denmark | 14.6 | 94.3 | 0.9 | 5.7 | 15.5 |
| Finland | 14.2 | 91.8 | 1.3 | 8.2 | 15.4 |
| Lithuania | 14.0 | 90.6 | 1.4 | 9.4 | 15.4 |
| Norway | 14.3 | 93.7 | 1.0 | 6.3 | 15.3 |
| Hungary | 14.3 | 96.3 | 0.6 | 3.7 | 14.8 |
| Russian Federation* | 12.7 | 86.5 | 2.0 | 13.5 | 14.7 |
| Montenegro* | 12.3 | 86.9 | 1.8 | 13.1 | 14.2 |
| Latvia | 12.2 | 90.9 | 1.2 | 9.1 | 13.4 |
| Slovenia | 12.3 | 93.1 | 0.9 | 6.9 | 13.2 |
| Sweden | 11.6 | 93.7 | 0.8 | 6.3 | 12.4 |
| Armenia* | 10.9 | 90.2 | 1.2 | 9.8 | 12.1 |
| Estonia | 10.8 | 91.9 | 0.9 | 8.1 | 11.8 |
| Germany§ | 11.6 | 99.9 | 0.0 | 0.1 | 11.7 |
| Kyrgyzstan* | 6.7 | 59.9 | 4.5 | 40.1 | 11.2 |
| Austria§ | 10.3 | 99.9 | 0.0 | 0.1 | 10.3 |
| Netherlands | 9.1 | 94.1 | 0.6 | 5.9 | 9.7 |
| Azerbaijan* | 6.9 | 77.6 | 2.0 | 22.4 | 8.9 |

*WHO Euro AMC Network

§Country for which hospital sector data were not included

**Table 4A. Patterns of consumption of antibacterials according to the AWaRe classification of antimicrobials, ESAC-Net, 2018**

| **Country** | **Category of the AWaRe classification** | | | |
| --- | --- | --- | --- | --- |
|  | **Access** | **Watch** | **Reserve** | **Unclassified** |
|  | **% of total consumption#** | | | |
| Iceland§ | 84.1 | 13.1 | <0.1 | 2.8 |
| Denmark | 79.2 | 18.9 | 0.1 | 1.8 |
| Netherlands | 72.4 | 27.3 | <0.1 | 0.2 |
| Finland | 71.7 | 20.7 | 0.1 | 7.6 |
| France | 71.0 | 28.8 | 0.2 | 0.1 |
| Sweden | 70.9 | 20.3 | 0.1 | 8.7 |
| Ireland | 68.2 | 31.4 | 0.3 | 0.1 |
| United Kingdom | 66.9 | 32.1 | 0.3 | 0.6 |
| Lithuania | 66.7 | 32.5 | 0.4 | 0.5 |
| Belgium | 66.2 | 33.7 | 0.1 | 0.1 |
| Portugal | 64.6 | 35.2 | 0.2 | 0.0 |
| Latvia | 63.7 | 31.7 | 0.1 | 4.5 |
| Spain | 62.8 | 36.7 | 0.3 | 0.2 |
| Croatia | 62.3 | 37.1 | 0.1 | 0.4 |
| Slovenia | 61.1 | 26.9 | 0.1 | 11.8 |
| Norway | 60.6 | 15.2 | 0.1 | 24.1 |
| Estonia | 60.6 | 39.3 | 0.1 | 0.0 |
| Austria§ | 58.2 | 40.2 | 0.1 | 1.5 |
| EU/EEA | 57.9 | 40.0 | 0.2 | 1.9 |
| Luxembourg | 57.7 | 42.1 | 0.1 | 0.1 |
| Germany§ | 56.1 | 42.9 | <0.1 | 1.0 |
| Romania | 53.1 | 46.5 | 0.2 | 0.1 |
| Cyprus | 48.6 | 51.3 | 0.1 | 0.1 |
| Hungary | 48.5 | 51.4 | <0.1 | 0.0 |
| Italy | 47.6 | 52.0 | 0.3 | 0.2 |
| Greece | 47.1 | 52.4 | 0.5 | 0.0 |
| Malta | 46.4 | 53.1 | 0.2 | 0.3 |
| Bulgaria | 44.9 | 55.0 | <0.1 | 0.1 |
| Poland | 43.9 | 44.2 | 0.1 | 11.9 |
| Slovakia | 39.1 | 60.7 | 0.1 | 0.1 |

AWaRe: Access, Watch and Reserve classification (World Health Organization 2019)

EU/EEA: population-weighted mean for countries of ESAC-Net

#agents included in this analysis: antibacterials for systemic use (J01), neomycin (A07AA01), streptomycin (A07AA04), polymyxin B (A07AA05), kanamycin (A07AA08), vancomycin (A07AA09), colistin (A07AA10), rifamixin (A07AA11), rifampicin (J04AB02), rifamycin (J04AB03), rifabutin (J04AB04), metronidazole (P01AB01).

§Country for which hospital sector data were not included

**Table 4B. Patterns of consumption of antibacterials according to the AWaRe classification of antimicrobials, WHO Europe AMC Network, 2018**

| **Country** | **Category of the AWaRe classification** | | | |
| --- | --- | --- | --- | --- |
|  | **Access** | **Watch** | **Reserve** | **Unclassified** |
|  | **% of total consumption#** | | | |
| Bosnia and Herzegovina | 65.8 | 33.5 | < 0.1 | 0.6 |
| Armenia | 62.5 | 35.6 | 0.1 | 1.8 |
| Azerbaijan | 60.9 | 35.5 | < 0.1 | 3.6 |
| Belarus | 57.4 | 36.6 | 0.7 | 5.3 |
| Montenegro | 57.3 | 35.5 | < 0.1 | 7.1 |
| Republic of Moldova | 51.4 | 46.1 | < 0.1 | 2.5 |
| Serbia | 51.0 | 45.8 | 0.1 | 3.1 |
| Kazakhstan | 50.7 | 45.2 | < 0.1 | 4.1 |
| Turkey | 50.2 | 47.5 | 0.1 | 2.3 |
| **WHO/AMC** | **47.6** | **49.1** | **0.1** | **3.2** |
| Russian Federation | 47.4 | 47.9 | 0.1 | 4.6 |
| Georgia | 43.2 | 54.2 | < 0.1 | 2.6 |
| Tajikistan | 42.1 | 55.5 | 0.1 | 2.3 |
| Albania | 39.4 | 60.1 | < 0.1 | 0.5 |
| Kyrgyzstan | 33.5 | 61.5 | 0.1 | 4.9 |
| Uzbekistan | 29.7 | 68.8 | < 0.1 | 1.4 |

AWaRe: Access, Watch and Reserve classification (World Health Organization 2019)

WHO/AMC: population-weighted mean for countries of WHO Europe AMC Network

#agents included in this analysis: antibacterials for systemic use (J01), neomycin (A07AA01), streptomycin (A07AA04), polymyxin B (A07AA05), kanamycin (A07AA08), vancomycin (A07AA09), colistin (A07AA10), rifamixin (A07AA11), rifampicin (J04AB02), rifamycin (J04AB03), rifabutin (J04AB04), metronidazole (P01AB01).
